# Arsenic trioxide inhibits lung metastasis of mouse colon cancer via reducing the infiltration of regulatory T cells

**DOI:** 10.1007/s13277-016-5377-3

**Published:** 2016-09-27

**Authors:** Lei Wang, Xiang Hu, Yingxin Xu, Zhong Liu

**Affiliations:** 1 Department of General Surgery, First Affiliated Hospital of Dalian Medical University, Dalian, 116011 China; 2 Zhongnan Hospital of Wuhan University, Institute of Hepatobiliary Disease of Wuhan University, Transplant Center of Wuhan University, Hubei Key Laboratory of Medical Technology on Transplantatation, Wuhan, 430071 China; 3 Institute of General Surgery, Chinese PLA General Hospital, Beijing, 100853 China; 4 Department of General Surgery, First Affiliated Hospital of Dalian Medical University, No. 222, Zhongshan Road, Dalian, 116011 China

**Keywords:** Arsenic trioxide, Colon cancer, Lung metastasis, Regulatory T cells, Cytokine-induced killer cells

## Abstract

The purpose of this study was to investigate the effects of arsenic trioxide (As_2_O_3_) on the infiltration of regulatory T cells (Tregs) in the local lung metastasis of mouse colon cancer in vivo and the regulation of Tregs in cytokine-induced killer cells (CIKs) in vitro. A high Tregs infiltration mouse colon cancer lung metastasis model was established by intravenous injection of CT26 murine colon carcinoma cells. Tumor-bearing mice were randomly divided into three groups: control group, low-dose As_2_O_3_ group, and high-dose As_2_O_3_ group. For in vitro studies, CIKs were treated with vehicle control or 0.1, 1, or 5 μM As_2_O_3_. The level of Tregs was detected via flow cytometry, Foxp3 expression was assessed by immunohistochemistry and reverse transcription–polymerase chain reaction (RT-PCR), the level of interferon gamma (IFN-γ) was evaluated by enzyme-linked immunoassay (ELISA), and the cytotoxic activity of As_2_O_3_-treated CIKs was assessed through a lactate dehydrogenase (LDH) release assay. Obvious lung metastasis was observed 3 days after CT26 murine colon carcinoma cell injection. The numbers of Tregs in the lungs and spleens of tumor-bearing mice were significantly higher than those of the normal group (*p* < 0.01). As_2_O_3_ treatment increased the mouse weight as well as reduced the number of metastatic lung nodules and the lung/body weight ratio (*p* < 0.01). Moreover, As_2_O_3_ treatment significantly reduced the Tregs proportion and the Foxp3 messenger RNA (mRNA) levels in metastatic lung tissues (*p* < 0.01). In vitro, As_2_O_3_ significantly reduced the Tregs proportion and the Foxp3 mRNA levels (*p* < 0.01) and significantly increased the cytotoxic activity of CIKs and the IFN-γ levels in the supernatant of cultured CIKs (*p* < 0.01). As_2_O_3_ might inhibit lung metastasis of colon cancer by reducing the local infiltration of Tregs and increase the cytotoxic activity of CIKs by suppressing Tregs.

## Introduction

Arsenic trioxide (As_2_O_3_) has been applied for the treatment of acute lymphoblastic leukemia. Emerging evidence has suggested that As_2_O_3_ has a therapeutic effect in many solid tumors by inducing apoptosis and inhibiting the invasion and migration of tumor cells [1–4]. Despite a limited anti-tumor effect of As_2_O_3_ monotherapy, it can enhance the anti-tumor effects of metformin [5] and sorafenib [6]. It has been well documented that immunity plays a key role in cancer therapy response [7]. Some studies have found that As_2_O_3_ can modulate the body’s immune response via CD4^+^ T/CD8^+^ T cells [8, 9], which suggests that As_2_O_3_ may be useful in tumor immunotherapy.

The number of regulatory T cells (Tregs) is highly increased in cancer patients, and Tregs promote tumorigenesis by reducing the number of T helper cells [10]. In addition, a high proportion of Tregs is one of the bottlenecks that affect the efficacy of adoptive immunotherapy [11]. Therefore, reducing the number of Tregs has become a critical issue for successful tumor immunotherapy. Cyclophosphamide, CD25 monoclonal antibodies, and cytotoxic T lymphocyte-associated protein 4 monoclonal antibodies have been shown to reduce the number of Tregs [12], but these drugs only transiently reduce the number of Tregs [13]. Hernandez et al. first reported that As_2_O_3_ could increase the Treg ratio in the spleen of myelitis rats [14]. Subsequently, Thomas et al. found that As_2_O_3_ reduced the proportion of Tregs in the spleen of a colon cancer subcutaneous rat model [15]. These findings suggest that As_2_O_3_ may act as a sensitizer of other therapeutic modules by reducing the number of Tregs. However, the molecular pharmacology of suppression of Tregs by As_2_O_3_ and whether As_2_O_3_ can reduce Tregs infiltration in the local metastatic tumor tissues remain to be determined.

In clinical practice, some colorectal cancer patients have lung metastases at diagnosis; moreover, lung metastasis also frequently occurs in colorectal cancer patients postoperation. It has been found that significantly increased numbers of tumor-infiltrating Tregs are seen in lung metastasis models of colon cancer as well as an increased proportion of Tregs in cytokine-induced killer cells (CIKs), so in this study, we investigated the effects of As_2_O_3_ on the infiltration of Tregs in local lung metastasis of mouse colon cancer in vivo and the regulation of Tregs in CIKs in vitro.

## Materials and methods

### Reagents and cell lines

As_2_O_3_ was purchased from Sigma (St. Louis, MO, USA) and stored at 4 °C. Human colon cancer SW-620 cells and mouse colon cancer CT26 cells were obtained from the Institute of General Surgery, Chinese PLA General Hospital (Beijing, China), and cultured in RPMI 1640 medium (Gibco, Grand Island, NY, USA), supplemented with 10 % inactivated fetal bovine serum (Gibco, Grand Island, NY, USA), 100 U/mL penicillin, and 100 μg/mL streptomycin at 37 °C in a 5 % CO_2_ incubator (Thermo Scientific, Waltham, MA, USA). Bouin’s solution was prepared by mixing 75 mL of saturated picric acid (Beijing Taiyu Tiangong Biotechnology, Beijing, China), 25 mL of 40 % formaldehyde, and 5 mL of acetic acid.

### Animal models and in vivo experiments

BALB/c female mice of 6–8 weeks old were purchased from the Beijing Experimental Animal Center of the Academy of Military Medical Sciences (Beijing, China). A total of 1 × 10^5^ mouse CT26 cells in 100 μL of phosphate-buffered saline (PBS) were slowly injected into mice through the tail vein to establish the mouse lung metastasis model of colon cancer. Three days after injection, tumor-bearing mice were randomly divided into three groups: control group (treated with saline, i.v.), low-dose group (treated with As_2_O_3_ at 3 mg/kg/day, i.v.), and high-dose group (treated with As_2_O_3_ at 6 mg/kg every other day, i.v.), and the treatment continued for 2 weeks. The weight of the mice was recorded every day. When the experiment was terminated, a mononuclear cell suspension was prepared from eyeball blood. Then, the lungs were removed with a sterile technique to calculate the lung/body weight ratio, and enriched lymphocytes were prepared from the left lungs (described below). A mononuclear cell suspension was prepared by grinding the spleen tissue. Aliquots of the peripheral blood, spleen fluid, and lung metastasis lymphocytes were analyzed by flow cytometry to determine the proportion of Tregs. Parts of the right lobe of the lungs were processed for hematoxylin and eosin (HE) staining, immunohistochemical staining of Foxp3, and reverse transcription–polymerase chain reaction (RT-PCR) analysis of Foxp3 messenger RNA (mRNA). Another batch of mice was used to evaluate the effects of As_2_O_3_ on the survival of the CT26 mice.

### Count of metastatic lung nodules

Lung tissues were first fixed and stained with Bouin’s solution, and then the metastatic lung nodules were classified under a microscope as described previously [16]. Metastatic nodule diameters of less than 0.5, 0.5–1, 1–2, and greater than 2 mm were classified as grade I, II, III, and IV metastasis, respectively. Then, the total number of metastases was calculated according to the following formula: total metastasis number = (grade I metastasis number) + (grade II metastasis number × 2) + (grade III metastasis number × 3) + (grade IV metastases number × 4).

### Enrichment of lymphocytes from lung tissues

The lungs were flushed three times with PBS in order to wipe off the lymphocytes resided in the branchial alveolus. Next, using sterile techniques, the left lung was removed, cut into pieces, and digested with collagenase II (Gibco, Grand Island, NY, USA), Dnase I (Gibco, Grand Island, NY, USA), and hyaluronic acid enzyme V (Sigma-Aldrich, St. Louis, MO, USA) for 3 h with magnetic stirring at room temperature. The mononuclear cell suspension was prepared by centrifugation (Eppendorf Minispin, Hamburg, Germany) and filtration, followed by discontinuous density gradient centrifugation with mouse lymphocyte separation medium (MP Biomedicals, Santa Ana, CA, USA). The lower layer of the cell suspension that was rich in lymphocytes was collected.

### HE staining and immunohistochemistry

The right lung was fixed with formalin, embedded in paraffin, and sliced into 4-μm sections. HE staining was performed to check for necrosis of the tumor cells. Infiltrating Foxp3^+^ Tregs in the local lung metastasis were analyzed by immunohistochemistry. Elivision immunohistochemistry was performed using the standard streptavidin peroxidase complex technique on 4-μm-thick paraffin-embedded tissue sections. After pretreatment and blocking, Foxp3 immunostaining was performed using a rabbit anti-mouse Foxp3 monoclonal antibody (Abcam, Cambridge, UK) as the primary antibody and streptavidin-biotinylated anti-rabbit immunoglobulin G as the secondary antibody. This procedure was followed by incubation with a streptavidin-horseradish peroxidase enzyme conjugate. The sections were visualized using 3,3′-diaminobenzidine and finally counterstained with hematoxylin. Negative controls were subjected to immunohistochemistry by replacing the primary antibody with PBS solution. Foxp3^+^ cells were stained tan or brown. The number of Foxp3^+^ cells in five random fields was counted under a light microscope at high magnification (×400, Olympus BX53, Japan). The average number of cells from five fields was defined as the number of Tregs.

### Preparation of human CIKs for in vitro experiments

A mononuclear cell suspension from 20 mL of healthy human peripheral blood was prepared by density gradient centrifugation with human lymphocyte separation medium (Chinese Academy of Medical Sciences, Beijing, China). After incubation for 3 h, the nonadherent floating cells were collected and cultured in 75-mm T-type flasks with 10 mL of Cellix 601 serum-free medium (Beijing Xin Ming Thai Biotechnology, Beijing, China) supplemented with 1000 U/mL recombinant human interferon gamma (IFN-γ) (R&D, MN, USA) at 37 °C in a humidified incubator containing 5 % CO_2_ for 24 h. The next day, 2 μg/mL anti-CD3 McAb (BD, NJ, USA) and 1000 U/mL recombinant human interleukin-2 (IL-2) (R&D, MN, USA) were added to the medium. Twenty-four hours later, the medium was replaced with fresh medium containing 1000 U/mL recombinant human IL-2. On day 5, the medium was replaced with Cellix 602 serum-free medium, and the cells were cultured in a 1000-mL culture bag. The cell status was frequently monitored, and media and recombinant human IL-2 were supplemented at appropriate times. The CIKs were harvested on day 14. For the in vitro studies, 2 × 10^7^ CIKs in a 100-mm dish were treated with vehicle control or 0.1, 1, or 5 μM As_2_O_3_ for 48 h. Then, the experiments were repeated three times in vitro. The ratio of CD4^+^ CD25^+^ Foxp3^+^ Tregs or CD4^+^/CD8^+^ T cells in the CIKs was determined by flow cytometry. The expression of Foxp3 in the CIKs was detected by RT-PCR. Enzyme-linked immunoassay (ELISA) was used to detect the content of IFN-γ in the cell culture supernatant. A lactate dehydrogenase (LDH) release assay was used to assess the cytotoxic activity of the CIKs.

### Flow cytometric detection of Tregs

The prepared CIKs were first incubated with CD4 FITC (BD, NJ, USA) and CD25APC (BD, NJ, USA), followed by permeabilization and staining with Foxp3-percp antibody (BD, NJ, USA), according to the manufacturer’s protocol. All samples were examined using a FACSCalibur instrument (BD, NJ, USA), and the data were analyzed using FlowJo 7.6.1 software.

### Cytotoxicity assay

The in vitro cytotoxicity of CIKs to SW-620 cells was performed by an LDH release assay (Sigma-Aldrich, St. Louis, MO, USA), as described previously [17]. The target SW-620 cells were mixed with CIKs treated with different concentrations of As_2_O_3_ at a ratio of 5:1 or 10:1 and cultured for 24 h. The optical density (OD) value was detected with a microplate reader at a wavelength of 492 nm. The killing rate was calculated as follows: Cytotoxicity % = [OD(Experimental) − OD(Effector spontaneous) − OD(Target spontaneous)] × 100 / [OD(Target maximum) − OD(Target spontaneous)].

### ELISA detection of cytokines

CIKs were treated with different concentrations of As_2_O_3_ for 48 h. The IFN-γ content in the supernatant was determined using an IFN-γ ELISA kit (Sigma-Aldrich, St. Louis, MO, USA), according to the manufacturer’s protocol. The OD value was detected with a microplate reader at a wavelength of 492 nm.

### RT-PCR detection of Foxp3

Mouse and human Foxp3 as well as β-actin primers were designed and synthesized by Shanghai Sangon (Shanghai, China). The sequences of the primers were as follows: mouse β-actin primer sequences, M-β-actin-F: 5ʹ-GTGCTATGTTGCTCTAGACTTCG-3ʹ; M-βactin-R: 5ʹ- ATGCCACAGGATTCCATACC-3ʹ; mouse Foxp3 primer sequence,

M-Foxp3-F: 5ʹ-TGGTTTACTCGCATGTTCGC-3ʹ, M-Foxp3-R: 5ʹ-CCCACCTTTTCTTGGTTTTGA-3ʹ’ human β-actin primer sequences, H-β-actin-F: 5ʹ-TAGTTGCGTTACACCCTTTCTTG-3ʹ, H-β-actin-R: 5ʹ-TCACCTTCACCGTTCCAGTTT-3ʹ; human Foxp3 primer sequence, H-Foxp3-F: 5ʹ-AGGAAAGGAGGATGGACGAA-3ʹ, H-Foxp3-R: 5ʹ-GCAGGCAAGACAGTGGAAAC-3ʹ. The lungs were flushed three times with PBS, and then the homogenates were prepared by rapidly grinding the frozen tissues in liquid nitrogen. ATO-treated CIKs were washed three times and resuspended at 1.0 × 10^7^/mL. Total RNA was extracted using TRIzol reagent (Life Technologies, Rockville, MD, USA), according to the manufacturer’s instructions. The amount of total RNA was determined by measuring the absorbance at 260 nm with a spectrophotometer. One microgram of total RNA was reverse-transcribed to the complementary DNA (cDNA) using the PrimeScript 1st Strand cDNA Synthesis Kit (Takara, Dalian, China), according to the manufacturer’s protocol. Real-time PCR was conducted by the SYBR Green method on a Corbett Rotor-Gene 3000 (Corbett Research, Concorde, Australia) real-time thermal cycler. Data were expressed using the comparative threshold cycle method and normalized to the housekeeping gene β-actin.

### Statistical analysis

SPSS 21.0 statistical software was used for statistical analysis of the relevant data. Data are expressed as the mean ± standard deviation. Differences between two groups were compared using the *t* test. Differences among several groups were analyzed using one-way analysis of variance. The Kaplan-Meier test was performed for survival analysis. *P* < 0.05 was considered statistically significant.

## Results

### Establishment of the mouse colon cancer lung metastasis model with high Treg infiltration

**Fig. 1:**
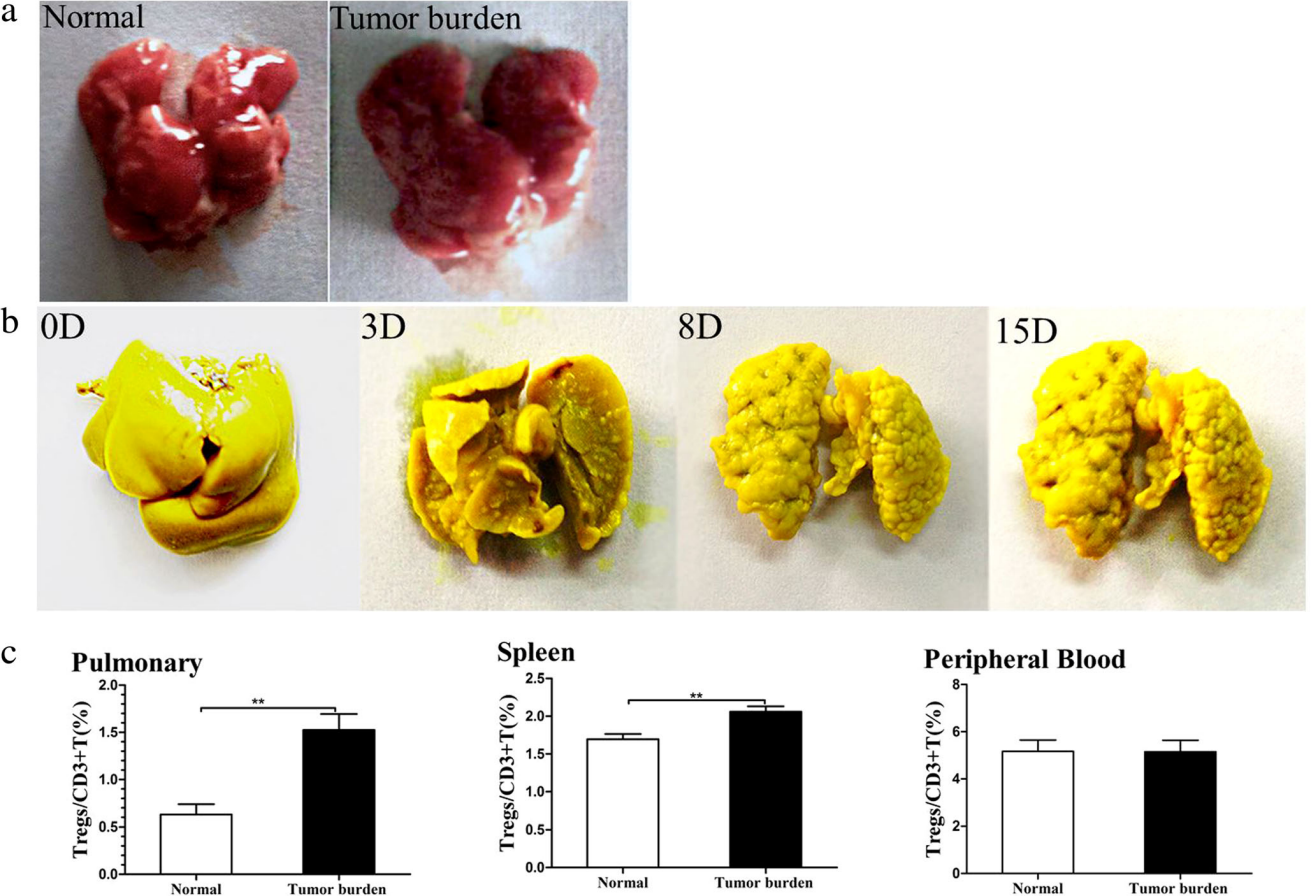
Establishment of the mouse colon cancer lung metastasis model with high Treg infiltration. Metastatic lung nodules appeared 3 days after intravenous injection of CT26 cells (**a**). The number and volume of nodules gradually increased and fused with time on days 0, 3, 8, and 15 (**b**). A significantly higher number of infiltrating Tregs in the lung and spleen of the model group was detected compared with that of the normal group (*p* < 0.01, C). However, there was no significant difference in the number of infiltrating Tregs in the peripheral blood between the model group and the normal group (**c**) (**p < 0.01, *n* = 6 per group)

BALB/c mice were randomly divided into two groups: normal group and model group. Metastatic lung nodules appeared 3 days after intravenous injection of CT26 cells (Fig. 1a). The number and volume of nodules gradually increased and fused with time (Fig. 1b). A significantly higher number of infiltrating Tregs in the model group was detected compared with that of normal group (*p* < 0.01, Fig. 1b). Moreover, the number of infiltrating Tregs in the spleen of the model group was also significantly higher than that of the normal group (*p* < 0.01, Fig. 1c). However, there was no significant difference in the number of infiltrating Tregs in the peripheral blood between model group and normal group (Fig. 1c).

### As_2_O_3_ inhibited lung metastasis of mouse colon cancer

**Fig. 2:**
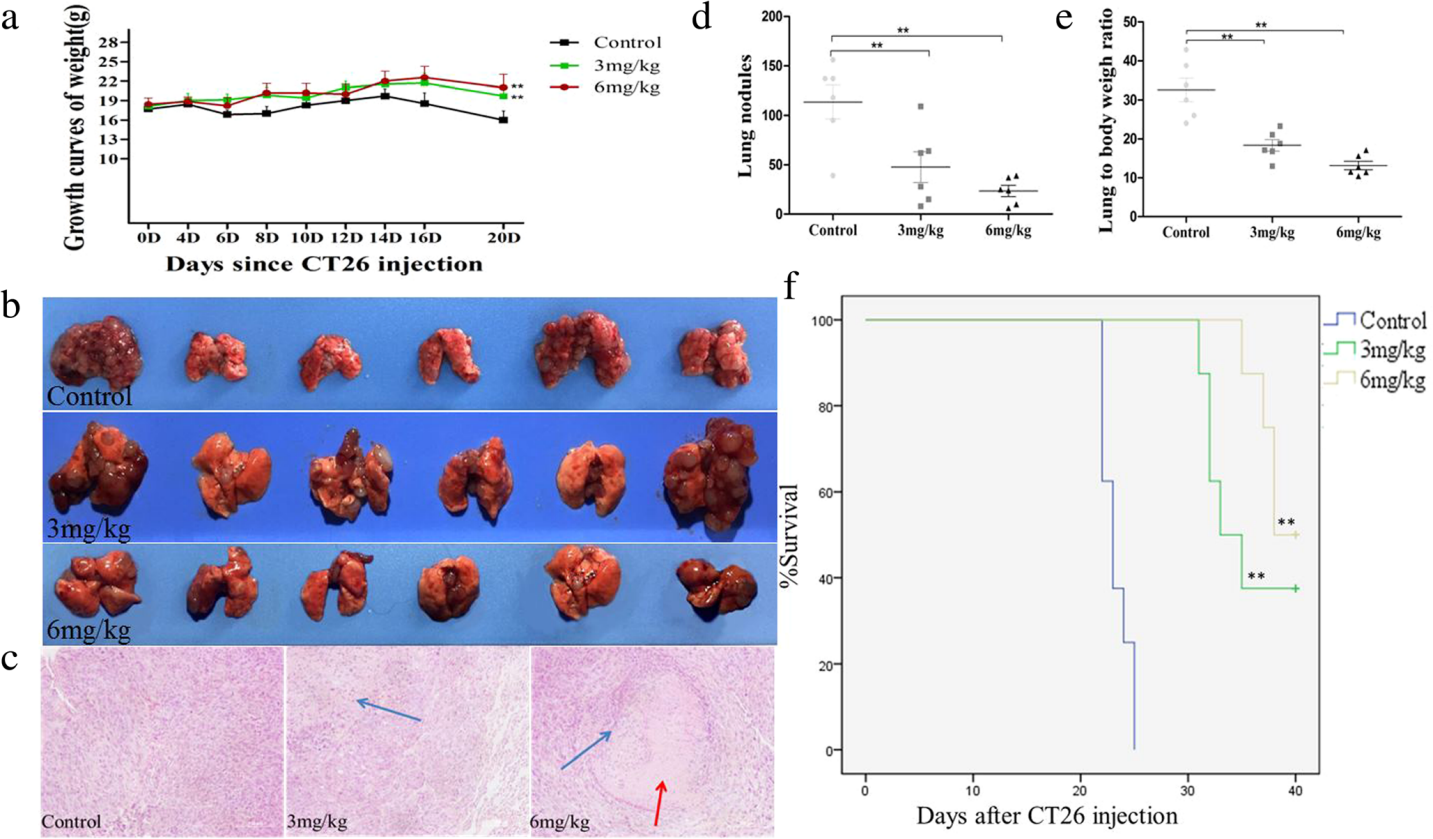
As_2_O_3_ inhibited lung metastasis of mouse colon cancer. After As_2_O_3_ treatment for 2 weeks, the mouse body weight increased significantly, with the most obvious effect in the high-dose group (*p* < 0.01); however, there was no significant difference of body weight between these two treatment groups (**a**). Physical analysis showed that both the lung volume and the number of metastatic lung nodules were decreased after As_2_O_3_ treatment (**b**). HE staining demonstrated increased tumor cell debris and tumor interstitial fibrosis in the As_2_O_3_ treatment groups, compared with the control group. The blue arrows represent cell debris, and the red arrow represents fibrosis (**c**). Quantitative analysis showed that As_2_O_3_ treatment significantly reduced the number of metastatic lung nodules (**d**) and the lung/body weight ratio (**e**), compared with the control group (**p < 0.01, *n* = 6 per group). The survival times of mice in the low- and high-dose groups were prolonged significantly (**p < 0.01, **f**), and the survival rates were 37.5 and 50 % at 40 days after CT26 injection, respectively (*n* = 8 per group, **f**)

Three days after the injection of CT26 cells, tumor-bearing mice were randomly divided into three groups: control group (treated with saline, i.v.), low-dose group (treated with As_2_O_3_ at 3 mg/kg/day, i.v.), and high-dose group (treated with As_2_O_3_ at 6 mg/kg every other day, i.v.). After As_2_O_3_ treatment, the mouse body weight increased significantly, with the most obvious effect in the high-dose group (*p* < 0.01). However, there was no significant difference of body weight between these two treatment groups (Fig. 2a). Physical analysis showed that both the lung volume and the number of metastatic lung nodules were decreased after As_2_O_3_ treatment (Fig. 2b). HE staining demonstrated increased tumor cell debris and tumor interstitial fibrosis in the As_2_O_3_ treatment groups, compared with the control group (Fig. 2c). Quantitative analysis showed that As_2_O_3_ treatment significantly reduced the number of metastatic lung nodules (Fig. 2d) and the lung/body weight ratio (Fig. 2e) (*p* < 0.01). The survival times of mice in the low- and high-dose As_2_O_3_ groups were significantly prolonged (*p* < 0.01, Fig. 2f), and the survival rates were 37.5 and 50 % at 40 days after CT26 injection, respectively (Fig. 2f).

### As_2_O_3_ reduced the infiltration of Tregs and suppressed the expression of Foxp3 in lung metastases of colon cancer in mice

**Fig. 3:**
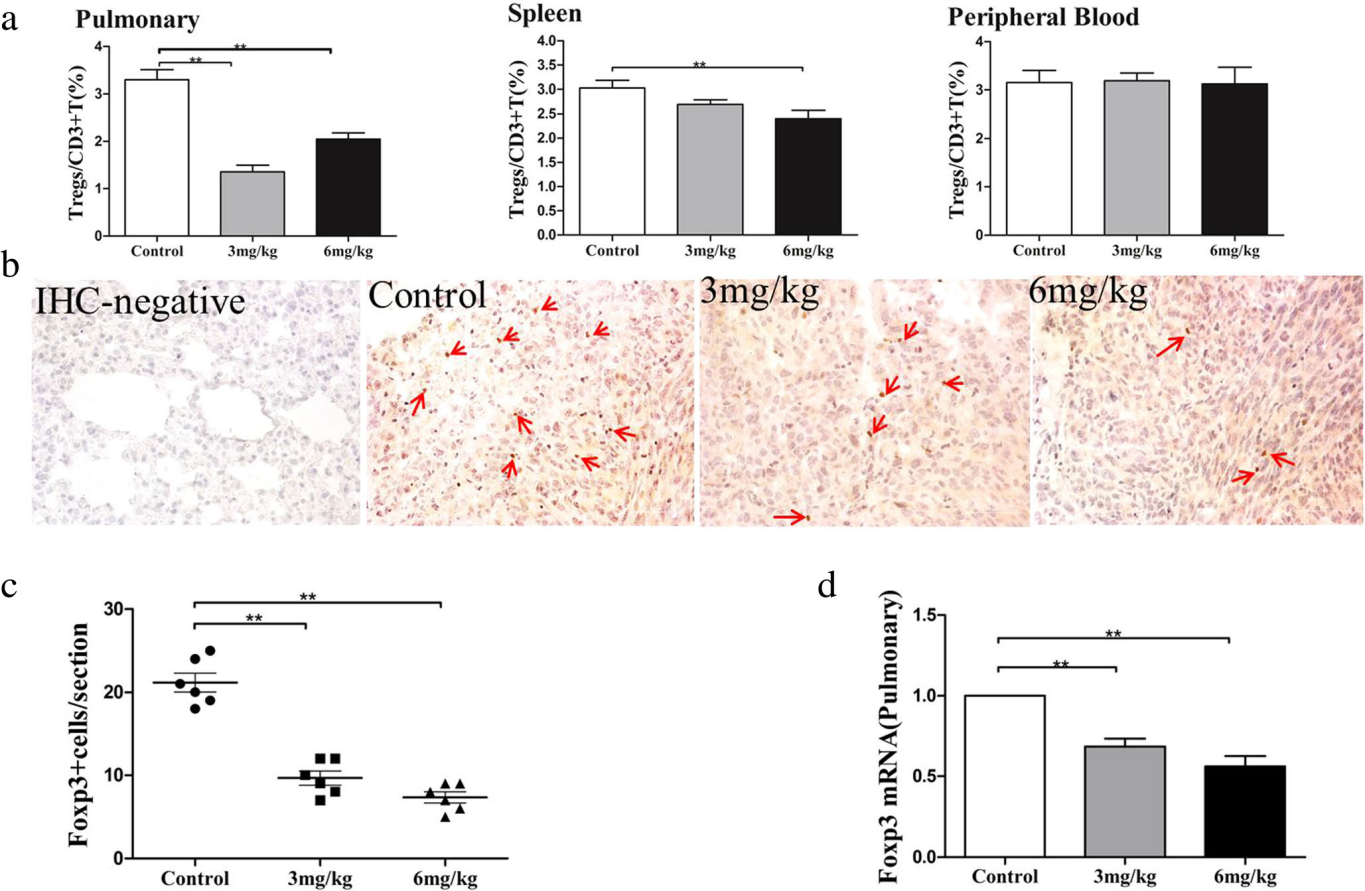
As_2_O_3_ reduced the infiltration of Tregs in lung metastases of mouse colon cancer. The flow cytometry results showed that both the low dose and the high dose of As_2_O_3_ significantly reduced the number of infiltrating Tregs in lung metastases, with a greater inhibitory effect by the low dose (*p* < 0.01, **a**). The high dose but not the low dose of As_2_O_3_ significantly reduced the number of infiltrating Tregs in the spleen (*p* < 0.01, **a**). Neither the low nor the high dose of As_2_O_3_ reduced the number of infiltrating Tregs in the peripheral blood (**a**). Immunohistochemical analysis showed lots of dark brown Foxp3+ staining in lung tissues before treatment, while in the low dose and the high dose of As_2_O_3_ groups, obviously reduced numbers of Foxp3_+_-stained cells in the lung were seen (**b**). Further semiquantitative analysis demonstrated that As_2_O_3_ treatment significantly decreased the number of Foxp3_+_ cells in the lung tissues (*p* < 0.01, **c**), but there was no significant difference between these two treatment groups (**c**). RT-PCR results showed the same trend, in which As_2_O_3_ treatment significantly decreased Foxp3 mRNA expression in the lung tissues (*p* < 0.01, **d**), and there was no significant difference between these two treatment groups (**d**). (**p < 0.01, *n* = 6 per group)

To test whether As_2_O_3_ reduces the infiltration of Tregs, the Treg ratios in the lung, spleen, and peripheral blood were assessed by flow cytometry. The results showed that both the low and high doses of As_2_O_3_ could significantly reduce the number of infiltrating Tregs in lung metastases, with a greater inhibitory effect by the low dose (*p* < 0.01, Fig. 3a). The high dose but not the low dose of As_2_O_3_ significantly reduced the number of infiltrating Tregs in the spleen (*p* < 0.01, Fig. 3a). Neither the low dose nor the high dose of As_2_O_3_ reduced the number of infiltrating Tregs in the peripheral blood (Fig. 3a). We determined the expression of Foxp3 in the lung by RT-PCR and immunohistochemistry. Immunohistochemical analysis showed lots of dark brown Foxp3^+^-stained cells in the lung tissues before treatment, while in the low-dose and the high-dose As_2_O_3_ groups, obviously reduced numbers of Foxp3^+^-stained cells in the lung were seen (Fig. 3b). Further semiquantitative analysis demonstrated that As_2_O_3_ treatment significantly decreased the number of Foxp3^+^ cells in the lung tissues (*p* < 0.01, Fig. 3c), but there was no significant difference between these two treatment groups (Fig. 3c). RT-PCR results showed the same trend, in which As_2_O_3_ treatment significantly decreased Foxp3 mRNA expression in the lung tissues (*p* < 0.01, Fig. 3d), and there was no significant difference between these two treatment groups (Fig. 3d).

### As_2_O_3_ dose-dependently decreased the percentage of Tregs in CIKs and improved the cytotoxic activity of CIKs in vitro

**Fig. 4:**
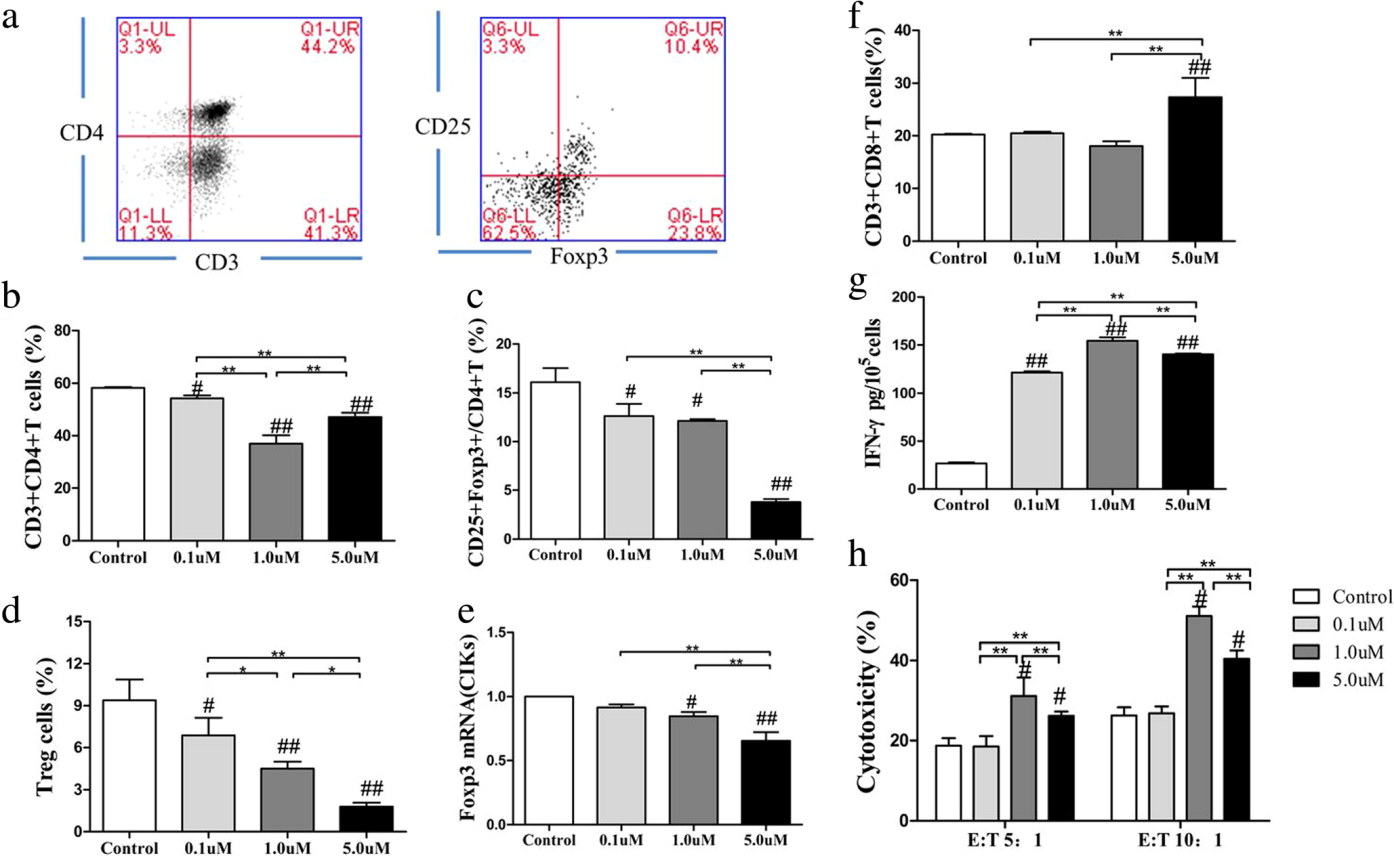
As_2_O_3_ reduced the Treg ratio in CIKs and improved the cytotoxic activity of CIKs in vitro. The expression of CD25 and Foxp3 was double-stained in the pooled CD4+ T cells after membranes rupture (**a**). The flow cytometry results showed that As_2_O_3_ treatment significantly decreased the percentage of CD4+ T cells, with a greater effect by 1 μM As_2_O_3_ (*p* < 0.01, **b**). As_2_O_3_ treatment significantly decreased the ratio of Tregs, with a greater effect by 5 μM As_2_O_3_ (*p* < 0.01, **c**). The expression of Foxp3 in the pooled CD4+ T cells was reduced in a dose-dependent manner (*p* < 0.01, **d**), which was confirmed with respect to the Foxp3 mRNA levels by RT-PCR (*p* < 0.01, **e**). The cytotoxic activity of As_2_O_3_-treated CIKs was evaluated through different ways in vitro. The ratio of CD8+ T cells increased significantly by treatment with 5 μM As_2_O_3_ (*p* < 0.05, **f**), and the level of IFN-γ harvested from the supernatant of CIKs increased significantly, with a greater effect by 1 μM As_2_O_3_ (*p* < 0.01, **g**). As_2_O_3_ treatment significantly improved the cytotoxic activity of CIKs at a ratio of 5:1 or 10:1 SW-620 cells to CIKs (*p* < 0.01, **h**) (# compared with the control group, #*p* < 0.05, ##*p* < 0.01; *compared between the experimental groups, *p < 0.05, **p < 0.01. The in vitro experiments were repeated three times)

To extend our in vivo observation and explore the potential pharmacology of As_2_O_3_ in reducing the tumor burden of the mouse colon cancer, we treated CIKs with different concentrations of As_2_O_3_ for 48 h. The expression of CD25 and Foxp3 was double-stained in the pooled CD4^+^ T cells (Fig. 4a). The flow cytometry results showed that As_2_O_3_ treatment significantly decreased the percentage of CD4^+^ T cells, with a greater effect by 1 μM As_2_O_3_ (*p* < 0.01, Fig. 4b). As_2_O_3_ treatment significantly decreased the ratio of Tregs, with a greater effect by 5 μM As_2_O_3_ (*p* < 0.01, Fig. 4c). Further studies showed that the expression of Foxp3 in the pooled CD4^+^ T cells was reduced in a dose-dependent manner (*p* < 0.01, Fig. 4d), which was confirmed with respect to the Foxp3 mRNA levels by RT-PCR (*p* < 0.01, Fig. 4e).

The cytotoxic activity of As_2_O_3_-treated CIKs was evaluated through different ways in vitro. First, the percentage of CD8^+^ T cells increased according to the different concentrations of As_2_O_3_, with a greater effect by 5 μM As_2_O_3_ (*p* < 0.05, Fig. 4f). Second, ELISA analysis detected a significant increase of IFN-γ levels in the CIK supernatants of the As_2_O_3_-treated groups (*p* < 0.01, Fig. 4g). Finally, we determined the in vitro cytotoxicity of CIKs toward SW-620 cells by the LDH release assay. The target SW-620 cells were mixed with As_2_O_3_-treated CIKs at a ratio of 5:1 or 10:1, and the cells were cultured for 24 h. The results showed that As_2_O_3_ treatment significantly improved the cytotoxic activity of CIKs (*p* < 0.01, Fig. 4h).

## Discussion

In this study, we demonstrated that As_2_O_3_ potently decreased the tumor burden and inhibited the lung metastasis of colon cancer in a mouse model. Importantly, we showed that As_2_O_3_ reduced the infiltration of Tregs and suppressed the expression of Foxp3 in lung metastases of colon cancer*.* In vitro, As_2_O_3_ significantly improved the cytotoxic activity of CIKs, accompanied with a decreased percentage of CD4^+^CD25^+^Foxp3^+^ Tregs in CIKs.

In the lung metastasis model of mouse colon cancer established in this study, lung metastasis appeared very early, with significant infiltration of Tregs/CD3 in the lung but not in the peripheral blood, suggesting an ideal model for the study of pharmacological inhibition of Tregs in the treatment of colon cancer. With this model, we found that As_2_O_3_ significantly inhibited the pulmonary metastasis of colon cancer, without significant toxicity. As_2_O_3_-treated mice displayed a significantly increased body weight and reduced the tumor burden and the number of metastatic lung nodules. However, no significant difference was observed between the low and high doses of As_2_O_3_. It has been reported that 6.5 mg/kg As_2_O_3_ is selectively enriched in tumor tissues rather than in the liver, brain, kidney, and other tissues and does not cause significant toxicity [18]. However, we found that in the first administration, high-dose As_2_O_3_ (6.0 mg/kg) induced transient acute toxicity in mice, which lasted for 10 min and returned to normal after 30 min (data not shown), suggesting that As_2_O_3_ toxicity in the first dosing should be noted. Moreover, As_2_O_3_ has been shown to induce tumor cell tolerance [19] and cumulative toxic effects [20], which may explain the similar effects of the low-dose and high-dose As_2_O_3_ treatment. Compared with 5-fluorouracil, As_2_O_3_ does not cause deterioration of the nutritional status of tumor-bearing mice [21]. Therefore, these facts suggest that As_2_O_3_ might be more effective than 5-fluorouracil for the treatment of lung metastasis in colon cancer.

Previous studies have shown that Tregs are selectively enriched in colon tumor sites and play a key role in tumor immune escape [22–24]. In addition, a high concentration of local tumor-infiltrating Tregs is the most crucial factor affecting the prognosis of patients with colon cancer [25–27]. Thus, reducing the number of local tumor-infiltrating Tregs may be of more clinical significance. Our in vivo study showed that As_2_O_3_ could significantly reduce local infiltration of Tregs and Foxp3 expression, which suggested that As_2_O_3_ inhibited lung metastasis of colon cancer via selectively reducing the infiltration of Tregs. In vitro, As_2_O_3_ not only selectively reduced the proportion of Tregs in CIKs but also increased the cytotoxic activity of CIKs, as demonstrated by the higher proportion of CD8^+^ T cells and the increased IFN-γ levels. Moreover, we found that As_2_O_3_ reduced the amount of CD4 ^+^ T cells, which was consistent with the previous reports [28, 29]. Taken together, As_2_O_3_ might inhibit lymphocyte proliferation largely through selective inhibition of CD4^+^ T cells, thereby increasing the CD8^+^ T cell ratio. Therefore, the appropriate dose of As_2_O_3_ might be used as an immune adjuvant to provide an improved treatment outcome for colon cancer.

The mechanism by which As_2_O_3_ reduces Tregs is unclear now. In this study, we found that the order of reduction of Foxp3 expression by As_2_O_3_ was high dose, medium dose, and low dose; the order of reduction of CD4^+^ T cells by As_2_O_3_ was medium dose, high dose, and low dose, which means that different concentrations of As_2_O_3_ reduce Tregs via different mechanisms: the medium concentration might mainly be via decreasing the proportion of CD4^+^ T cells, while the high concentration might mainly be through reducing Foxp3 expression. Furthermore, the detailed mechanisms need to be explored in the future.

In summary, we demonstrated that As_2_O_3_ might inhibit lung metastasis of colon cancer by reducing the local infiltration of Tregs and increase the cytotoxic activity of CIKs by suppressing Tregs.
